# Saikosaponin D Inhibits Lung Metastasis of Colorectal Cancer Cells by Inducing Autophagy and Apoptosis

**DOI:** 10.3390/nu16121844

**Published:** 2024-06-12

**Authors:** Yoon-Seung Lee, Jeong-Geon Mun, Shin-Young Park, Dah Yun Hong, Ho-Yoon Kim, Su-Jin Kim, Sun-Bin Lee, Jeong-Ho Jang, Yo-Han Han, Ji-Ye Kee

**Affiliations:** 1 Department of Oriental Pharmacy, College of Pharmacy, Wonkwang-Oriental Medicines Research Institute, Wonkwang University, 460 Iksandae-ro, Iksan 54538, Jeonbuk, Republic of Korea; 2 Department of Microbiology, Wonkwang University School of Medicine, Iksan 54538, Jeonbuk, Republic of Korea

**Keywords:** apoptosis, autophagy, colorectal cancer, metastasis, saikosaponin D

## Abstract

Saikosaponin D (SSD), derived from *Bupleurum falcatum* L., has various pharmacological properties, including immunoregulatory, anti-inflammatory, and anti-allergic effects. Several studies have investigated the anti-tumor effects of SSD on cancer in multiple organs. However, its role in colorectal cancer (CRC) remains unclear. Therefore, this study aimed to elucidate the suppressive effects of SSD on CRC cell survival and metastasis. SSD reduced the survival and colony formation ability of CRC cells. SSD-induced autophagy and apoptosis in CRC cells were measured using flow cytometry. SSD treatment increased LC3B and p62 autophagic factor levels in CRC cells. Moreover, SSD-induced apoptosis occurred through the cleavage of caspase-9, caspase-3, and PARP, along with the downregulation of the Bcl-2 family. In the in vivo experiment, a reduction in the number of metastatic tumor nodules in the lungs was observed after the oral administration of SSD. Based on these results, SSD inhibits the metastasis of CRC cells to the lungs by inducing autophagy and apoptosis. In conclusion, SSD suppressed the proliferation and metastasis of CRC cells, suggesting its potential as a novel substance for the metastatic CRC treatment.

## 1. Introduction

Colorectal cancer (CRC) affects the colon and rectum, which are vital organs of the digestive system. It usually originates from adenomatous polyps and abnormal growth in the colon or rectum. Globally, it is the third most common cancer, accounting for 10.0% of all cancer cases, and the second leading cause of cancer-related deaths at 9.4% [1]. In South Korea, CRC has the second highest incidence rate and is the third leading cause of mortality, following lung and liver cancer [2]. CRC with distant metastasis is included in stage IV, and colon cancer cells in this phase mainly spread to the liver and lungs [3,4]. Approximately 25% of patients with CRC are initially diagnosed with distant metastatic CRC and 50% of them develop metastatic disease [5]. According to the National Cancer Institute, as CRC can spread to distant organs, the 5-year survival rate of patients with CRC is only 14% [6].

Apoptosis and type I programmed cell death involve both intrinsic and extrinsic pathways [7]. In the intrinsic pathway, known as the mitochondrial pathway, cytochrome C is released from the mitochondria as a result of the suppression of the anti-apoptotic Bcl-2 family proteins [8,9]. After the activation of caspase-9 by cytochrome C, caspase-3 and poly (ADP-ribose) polymerase (PARP) are cleaved, leading to cell apoptosis [10,11]. The extrinsic pathway begins with the binding of death ligands to cell surface death receptors and activated caspase-8 influences caspase-3 cleavage [12,13,14]. PARP is cleaved by the activation of caspase-3 [15]. Apoptosis occurs when caspase-3 and PARP are cleaved in response to various stimuli.

Autophagy is a type II programmed cell death process. It is a process by which cells eliminate cellular waste, degenerated proteins, and malfunctioning organelles through self-devouring. These target components are recycled to generate energy required for cell survival or to create new cellular organelles [16]. These substances are isolated from the double-membrane autophagosomes [17]. The vesicles fuse with lysosomes to form autolysosomes, which are then degraded by lysosomal enzymes [18].

In traditional oriental medicine, *Bupleurum falcatum* L. is used to treat fever, inflammation, and pain [19,20]. Saikosaponins derived from the roots of *Bupleurum falcatum* L. are categorized into nine types: A–I [21]. Among them, saikosaponin D (SSD) has many pharmacological activities, including immunoregulatory, anti-inflammatory, and anti-allergic effects [22,23,24]. Several studies have investigated the anti-cancer effects of SSD on lung, liver, breast, prostate, pancreatic, and cervical cancers [25,26,27,28,29]. However, the suppressive effects of SSD on CRC progression have not yet been reported. Therefore, this study aimed to demonstrate that SSD inhibited the survival and lung metastasis of CRC cells through in vitro and in vivo experiments.

## 2. Materials and Methods

### 2.1. Reagents and Antibodies

SSD (purity > 95%) was purchased from Chengdu Biopurify Phytochemicals, Ltd. (Chengdu, China). Anti-p-p38, p-ERK, p-JNK, p-AMPK, AMPK, PARP, cleaved PARP, caspase-3, cleaved caspase-3, caspase-9, cleaved caspase-9, Bcl-2, Bcl-xL, cytochrome C, LC3B, and p62 antibodies were purchased from Cell Signaling Technology, Inc. (Danvers, MA, USA). Anti-p38, ERK, JNK, GAPDH, and β-actin antibodies were purchased from Santa Cruz Biotechnology, Inc. (Santa Cruz, CA, USA).

### 2.2. Cell Culture

Murine-derived CRC cell lines (CT26 and MC38) were cultured in Dulbecco’s Modified Eagle’s medium (Gibco BRL, Grand Island, NY, USA). Human CRC cell lines (HCT116 and SW620) were cultured in RPMI Medium 1640 (Gibco, Grand Island, NY, USA). Cell culture media were supplemented with 10% heat-inactivated fetal bovine serum and 1% penicillin–streptomycin (Gibco BRL). Cells were incubated at 37 ℃ in a 5% CO_2_ atmosphere.

### 2.3. Cell Viability Assay

CT26 and MC38 cells (1 × 10^4^ cells/well) and HCT116 and SW620 cells (2 × 10^4^ cells/well) were seeded into 48-well culture plates. After overnight incubation, cells were treated with SSD (2.5, 5, and 10 μM) for 24 h. The media was then removed and replaced with EZ-cytox (WST reagent, Donginbiotech Co., Seoul, Republic of Korea) mixed media. Following a 2 h incubation, the color transition was detected using a microplate reader at 450 nm.

### 2.4. Colony Formation Assay

CT26 (5 × 10^2^ cells/well) and HCT116 (1 × 10^3^ cells/well) cells were seeded into 12-well culture plates and incubated overnight. Cells were treated with SSD (2.5, 5, and 10 μM) for 7 days. After removing the media, 10% formaldehyde was added to the wells to fasten the colonies. The colonies were stained with 0.4% crystal violet solution (Sigma-Aldrich, St. Louis, MO, USA).

### 2.5. Autophagy Detection

CT26 and HCT116 cells (2 × 10^3^ cells/well) were seeded into 96-well culture plates and incubated with SSD for 48 and 72 h, respectively. The Muse™ Autophagy LC3-antibody-based kit (Luminex, Austin, TX, USA) was utilized to determine the induction of autophagy. Experiments were performed following the manufacturer’s protocol. The fluorescence of LC3 in autophagosomes was analyzed using a Muse™ Cell Analyzer.

### 2.6. Annexin V Assay

CT26 (1 × 10^4^ cells/well) and HCT116 (2 × 10^4^ cells/well) cells were incubated into a 48-well culture plate and treated with SSD for 24 h. The cells were then collected and resuspended in a fresh serum-containing medium. The Muse™ Annexin V & Dead Cell Reagent (Luminex) was added to 100 μL of the cells in suspension. The samples were then incubated for 20 min in the dark. Annexin V-positive cells were detected using a Muse Cell Analyzer.

### 2.7. Detection of Mitochondrial Membrane Potential

CT26 (3 × 10^5^ cells/well) and HCT116 (5 × 10^5^ cells/well) cells were seeded into 6-well culture plates and cultured with SSD for 24 h. Subsequently, the cells were treated with 1X assay buffer. The cells in suspension (100 μL) were mixed with 95 μL of MitoPotential working solution (Luminex) and incubated in a 37 ℃ CO_2_ incubator for 20 min. Five microliters of Muse MitoPotential 7-AAD reagent (Luminex) was added and the mixture was incubated for 5 min. The Muse™ Cell Analyzer was used to measure the depolarization of the mitochondrial membrane potential.

### 2.8. Western Blot Analysis

CT26 and HCT116 (3 × 10^5^ cells/well) cells were seeded into 6-well culture plates. SSD was then added to the cells for 24 and 48 h, respectively. To confirm the signaling pathway, CT26 (5 × 10^5^ cells/well) and HCT116 (1 × 10^6^ cells/well) cells were seeded into a 6-well culture plate and treated with SSD for 30 min. Ice-cold PRO-PREP protein extraction solution (iNtRon Biotechnology, Seongnam-si, Gyeonggi-do, Republic of Korea) was added to lyse the cells. The harvested lysates were vortexed every 15 min for 45 min and centrifuged at 13,000 rpm for 15 min at 4 °C to separate the supernatant from the pellet. Proteins were quantified using the bicinchoninic acid assay. The lysates were mixed with 2X buffer (Bio-Rad Laboratories, Hercules, CA, USA) for 5 min at 95 °C. Equal amounts of protein were loaded into the wells of an SDS-polyacrylamide gel and electrophoresed for 2 h 30 min. The proteins were transferred onto methylated PVDF membranes. EveryBlot blocking buffer (Bio-Rad Laboratories) was used to block the membranes for 10 min. After washing with 0.1% Tween 20 in TBS (TBST), the membranes were incubated overnight with primary antibodies. After overnight incubation, the membranes were washed with 0.1% TBST for 45 min. The membranes were incubated with secondary antibodies for 50 min. Subsequently, the membranes were washed with 0.1% TBST for 2 h. For protein expression detection, the D-Plus^TM^ ECL Pico system (Dongin LS, Seoul, Republic of Korea) and the FluorChem M System (ProteinSimple, San Jose, CA, USA) were used to visualize protein bands.

### 2.9. Real-Time Reverse Transcription-Polymerase Chain Reaction (RT-PCR)

The RNA-spin^TM^ Extraction kit (iNtRon Biotech, Seoul, Republic of Korea) and iScrpit cDNA Synthesis kit (Bio-Rad Laboratories) were used to extract total RNA and synthesize cDNA, respectively. All experiments were conducted according to the manufacturer’s protocol. CT26 (3 × 10^5^ cells/well) and HCT116 (5 × 10^5^ cells/well) cells were cultured with SSD for 24 h and harvested. The cell culture medium was completely removed and lysis buffer mixed with 70% ethanol was added to the cell membrane lysate. The cell lysates were centrifuged at 13,000 rpm for 30 s to remove the pellet. Washing buffers A and B were added sequentially and the cells were centrifuged under the same conditions. The cell lysate was centrifuged at 13,000 rpm for 2 min to dry RNA spin membranes. Elution buffer was directly added to the membrane for 5 min and spun down for 1 min at 13,000 rpm. The expression of target genes with the Real-time qPCR 2X Master Mix (ElpisBiotech, Daejeon, Republic of Korea) was quantified using a StepOnePlus^TM^ Real-time PCR System (Applied Biosystems by Thermo Fisher Scientific, Waltham, MA). The primer sequences are illustrated in Table S1. The mRNA expression levels of these genes were normalized to that of GAPDH.

### 2.10. Animal Care and Model of Lung Metastasis

Female BALB/c mice aged 6 weeks were purchased from Samtako (Osan, Republic of Korea) and allowed to adapt for one week before the start of the experiment. The mice were randomly divided into cages in the five groups. Six mice per group were required for this experiment based on power analysis [30]. CT26 cells (1 × 10^5^ cells) were mixed with 100 μL of PBS and injected into the tail vein of mice (i.v.). SSD (5, 10, and 20 mg/kg) was administered orally once daily for 14 days. As a positive control, 5-FU (30 mg/kg) was injected intraperitoneally twice a week during the same period. After 2 weeks, the lung tissues were excised and stained using Bouin’s solution (Sigma-Aldrich). The nodule numbers in the lungs were counted to determine the effect of SSD on metastatic CRC. In the case where a mouse’s body weight decreased by more than 20%, it was scheduled for euthanasia. All of the data were double-blind tested. In vivo experiments were performed in compliance with the accepted international principles for the handling and care of laboratory animals, as specified in the Wonkwang University guidelines (WKU23-23), and were under the ARRIVE guidelines.

### 2.11. Statistical Analysis

The results are presented as mean ± standard deviation. Statistical analyses were conducted using the Student’s *t*-test and one-way ANOVA to assess differences between groups. All statistical analyses were performed using SPSS Statistics v18 (IBM Corporation, Armonk, NY, USA). *p < 0.05, **p < 0.01, and ***p < 0.001 were considered to indicate a statistically significant difference.

## 3. Results

### 3.1. SSD Suppressed The Proliferation and Colony Formation of CRC Cells

**Figure 1:**
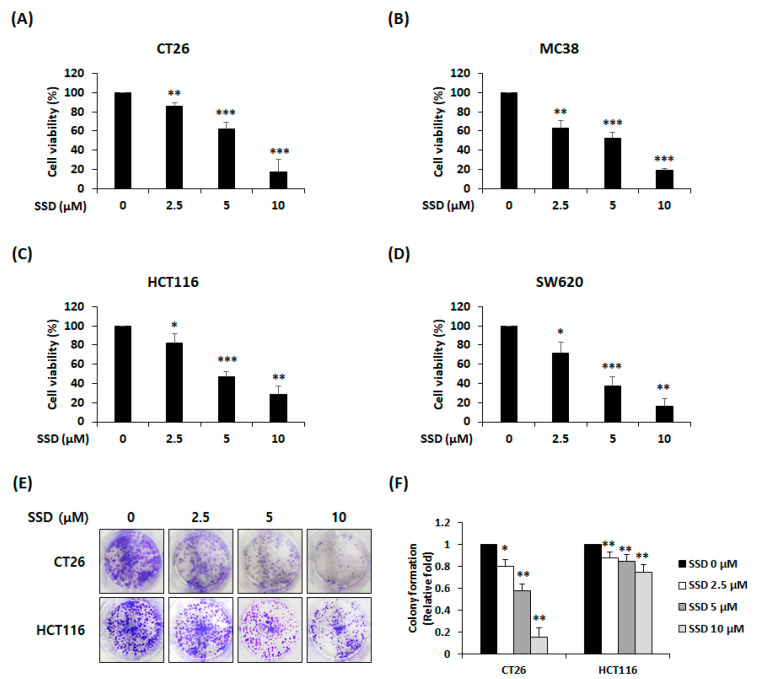
Effect of SSD on the proliferation and colony formation of CRC cells. (**A**–**D**) Viability of SSD-treated CT26 (**A**), MC38 (**B**), HCT116 (**C**), and SW620 (**D**) cells. The cells were treated with SSD (2.5, 5, and 10 μM) for 24 h. Cell viability was determined using the WST reagent. (**E**) Colony formation of CT26 and HCT116 cells after SSD treatment for 7 days. (**F**) Relative level of colony formation was measured using a microplate reader. The results are presented as the mean ± standard deviation of three repeated experiments. * *p* < 0.05, ** *p* < 0.01, and *** *p* < 0.001.

The WST assay was conducted to assess the viability of SSD-treated CRC cells. CT26, MC38, HCT116, and SW620 cells were treated with SSD (2.5–10 µM) for 24 h. SSD treatment led to a dose-dependent decrease in the cell survival rates (Figure 1A–D). The viability at the highest concentration (10 µM) was 17.5%, 19.3%, 28.2%, and 16.3% in CT26, MC38, HCT116, and SW620 cells, respectively. In addition, the half maximal inhibitory concentration (IC50) of CT26, MC38, HCT116, and SW620 cells was 6.40 ± 0.83 µM, 5.50 ± 0.18 µM, 6.35 ± 0.60 µM, and 5.14 ± 0.67 µM, respectively. The colony formation assay was performed to confirm the effect of SSD on the ability of CRC cells to form colonies. SSD treatment suppressed colony formation (Figure 1E). In particular, at 10 µM SSD, the colony formation ability of CT26 and HCT116 cells was reduced to 14.9% and 74.1%, respectively (Figure 1F).

### 3.2. SSD-Induced Autophagy in CRC Cells

**Figure 2:**
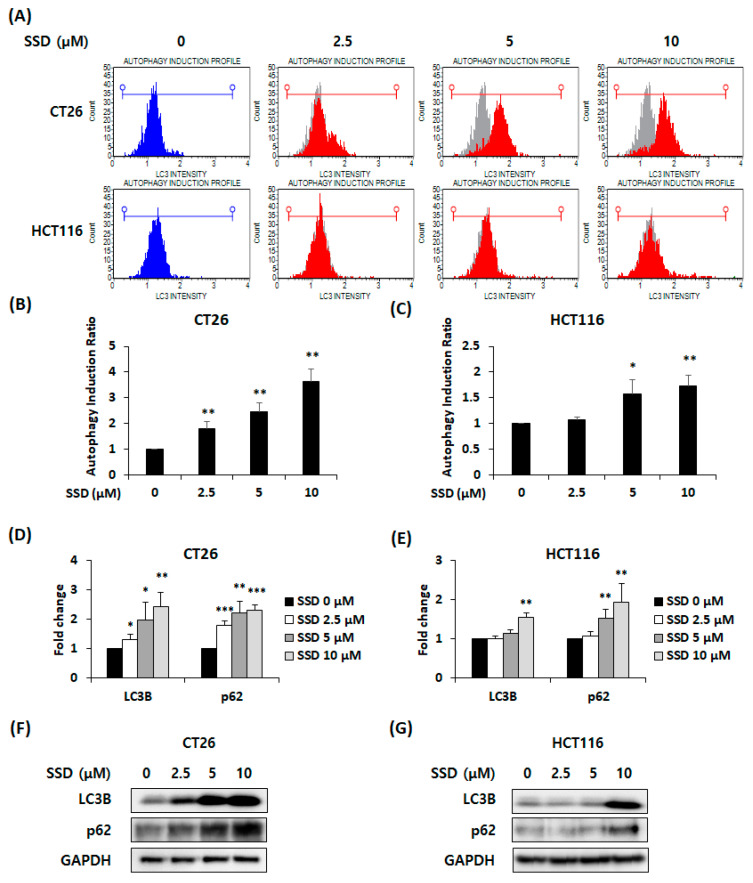
Effect of SSD on LC3B and p62-mediated autophagy in CRC cells. (**A**) LC3 detection in CT26 and HCT116 cells treated with SSD for 48 h using flow cytometry. (**B**,**C**) Autophagy induction ratio in CT26 (**B**) and HCT116 (**C**) cells. (**D**,**E**) mRNA expression of LC3B and p62 was measured using real-time RT-PCR. (**F**,**G**) Protein levels of autophagic factors were detected using Western blot analysis. The results are presented as mean ± standard deviation of three repeated experiments, and the representative images are presented. *p < 0.05, ** *p* < 0.01, and *** *p* < 0.001.

To determine whether SSD-induced autophagy was present in CRC cells, we performed flow cytometry and analyzed the autophagy induction ratio in SSD-treated CRC cells. The autophagy induction ratio of cells was 3.63-fold and 1.73-fold in 10 μM of SSD-treated CT26 and HCT116 cells, respectively (Figure 2A–C). Real-time RT-PCR and Western blot analyses were conducted to detect the mRNA and protein expression of autophagic factors LC3B and p62 in SSD-treated CRC cells. The mRNA levels of these autophagic factors were increased by SSD treatment (Figure 2D,E). LC3B and p62 protein expressions were also increased in SSD-treated CRC cells (Figure 2F,G). Therefore, SSD exhibited autophagy in CRC cells by upregulating LC3B and p62 expression.

### 3.3. SSD Triggered Apoptosis of CRC Cells by Activating the Mitogen-Activated Protein Kinase (MAPK) Signaling Pathway

**Figure 3:**
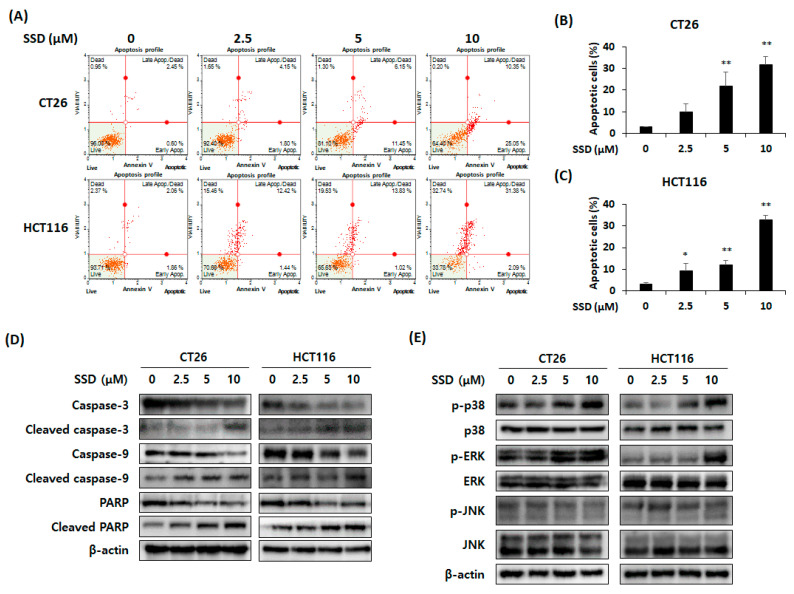
Effect of SSD on apoptosis of CRC cells through intrinsic and extrinsic pathways. (**A**) Annexin V assay of CRC cells after SSD treatment for 48 h. (**B**,**C**) Percentage of apoptotic cells in SSD-treated CT26 (**B**) and HCT116 (**C**) cells. (**D**) CT26 and HCT116 cells were incubated in 6-well culture plates and treated with SSD for 24 h. Apoptosis-related proteins were determined using Western blot analysis. (**E**) Protein levels of MAPKs in SSD-treated CT26 and HCT116 cells for 30 min were measured using Western blot analysis. The results are presented as mean ± standard deviation of three repeated experiments, and the representative images are included. * *p* < 0.05 and ** *p* < 0.01.

An annexin V assay was performed to identify other causes for the decreased survival rate of CRC cells following SSD treatment. The percentage of apoptotic cells increased by approximately 30% with 10 μM of SSD in CT26 and HCT116 cells (Figure 3A–C). Changes in the expression of apoptotic and signaling factors were measured in SSD-treated CRC cells. The SSD treatment of CT26 and HCT116 cells resulted in the cleavage of caspase-9 and caspase-8, which are related to the intrinsic and extrinsic pathways, respectively. These proteins influenced caspase-3 activation. As a result, caspase-3 and PARP were cleaved after SSD treatment (Figure 3D). To identify the signaling pathway that influenced the decrease in the proliferation of CRC cells, the activity of MAPKs was confirmed by Western blot analysis. CT26 and HCT116 cells were treated with SSD for 30 min. SSD-treated CT26 and HCT116 cells showed an increase in the activation of p38 and ERK, but the expression of JNK did not change (Figure 3E). Based on these results, SSD may decrease cell proliferation via the p38 and ERK signaling pathways.

### 3.4. SSD Increased Apoptosis of CRC Cells via Mitochondrial Depolarization

**Figure 4:**
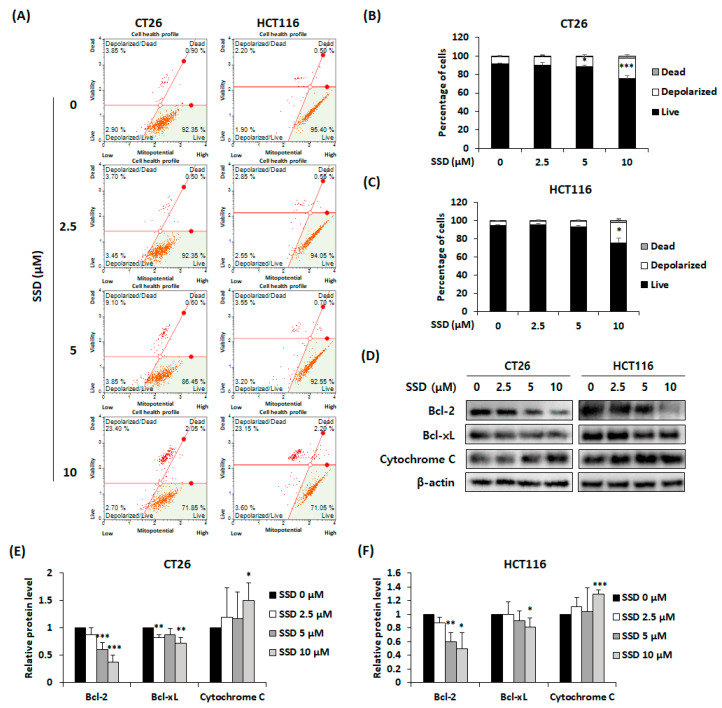
Effect of SSD on mitochondrial depolarization of CRC cells. (**A**) Mitochondrial assay of CT26 and HCT116 cells after SSD treatment for 24 h. (**B**,**C**) Percentage of depolarized cells in SSD-treated CT26 (**B**) and HCT116 (**C**) cells. (**D**) CT26 and HCT116 cells were treated with SSD for 24 h. The protein expression of mitochondria-associated apoptosis factors was detected using Western blot analysis. (**E**,**F**) Image J software (1.52a) was used to measure relative protein levels. The results are presented as mean ± standard deviation of three repeated experiments, and the representative images are included. * *p* < 0.05, ** *p* < 0.01 and *** *p* < 0.001.

A mitopotential assay was conducted to observe changes in the depolarization of the mitochondrial membrane potential. SSD treatment increased mitochondrial depolarization in CT26 and HCT116 cells (Figure 4A–C). Protein levels of intrinsic apoptosis-related factors were confirmed by Western blot analysis. The anti-apoptotic proteins Bcl-2 and Bcl-xL were downregulated, whereas the release of cytochrome C was increased by SSD (Figure 4D–F). These results demonstrated that treatment with SSD induces mitochondria-mediated apoptosis.

### 3.5. SSD Reduced Lung Metastasis of CRC Cells through Apoptosis and Autophagy

**Figure 5:**
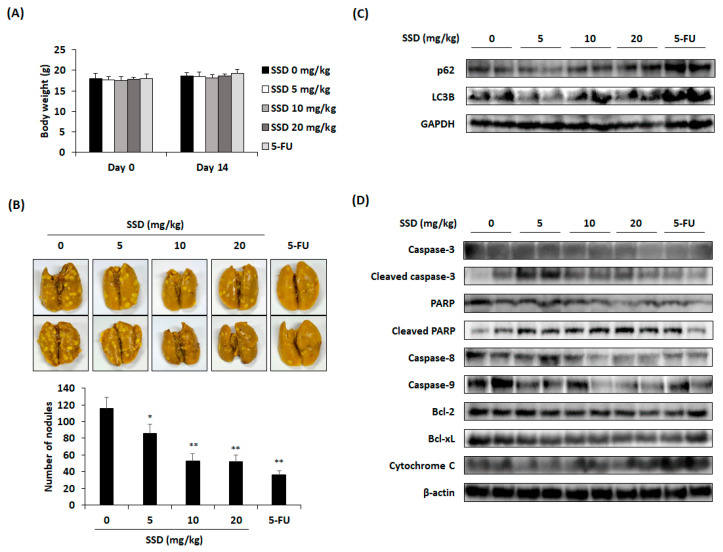
Effect of SSD on lung metastasis of CT26 cells in mouse model (*n* = 6 per group). (**A**) Body weight of mice for 14 days. (**B**) Representative images of the lungs fixed with Bouin’s solution and the number of nodules in the lungs. (**C**,**D**) Representative results of Western blot analysis of autophagic protein expression (**C**) and apoptosis-related protein expression (**D**) in the lung tissues. The SSD non-treated group (0 mg/kg) was used as a control group and compared with the SSD-treated group control group. The results are presented as mean ± standard deviation. * *p* < 0.05 and ** *p* < 0.01.

To elucidate the effect of SSD on CRC cells, we established a CT26 cell-injected lung metastasis mouse model. After injecting CT26 cells intravenously, SSD was administered orally and 5-FU was injected intraperitoneally for 14 days. Based on the serum analysis, there was clear evidence that SSD did not cause hepatotoxicity or nephrotoxicity (Table 1). In addition, there was no significant change in the body weight of the SSD-administered mice compared with that in the control group (Figure 5A). Oral administration of SSD reduced the number of tumor nodules in the lungs (Figure 5B,C). The protein levels of autophagy- and apoptosis-related factors in lung tissues were assessed by Western blot analysis. SSD increased the expression of autophagy-related factors including LC3B and p62 (Figure 5C). It also promoted the cleavage of PARP, caspase-3, caspase-8, and caspase-9, and the release of cytochrome C, while downregulating the Bcl-2 family (Figure 5D). These data suggested that SSD suppresses the lung metastasis of CT26 cells by inducing autophagy and apoptosis.

## 4. Discussion

Approximately half of CRC patients experience a significant advancement of the disease, reaching stage IV [5]. Metastatic CRC is typically treated with chemotherapy, surgery, or radiotherapy. However, these procedures can also lead to additional side-effects, including hair loss, nausea, vomiting, and diarrhea. Surgery and radiation therapy also impose strain on the body, causing infections or skin irritation at target sites [31]. Owing to the limitations of the existing treatments, there is a demand for new therapeutic approaches. Among these, ingredients from natural products can be used as potential solutions. For example, curcumin, extracted from *Curcuma longa* L., has the potential to prevent and treat CRC by modulating the gut microbiome, alleviating intestinal inflammation, and enhancing intestinal barrier function [32]. Natural compounds, such as wogonin, emodin, and ligustrazine, help in the prevention and treatment of CRC [33].

*Bupleurum falcatum* L. is used as a traditional herbal medicine in East Asia to treat diverse symptoms such as inflammation and pain [19]. In oriental medicine theory, one of the causes of cancer development is fever and toxins, which are similar to inflammatory factors [34]. Thus, the removal of fever is considered an effective approach for cancer treatment. In this sense, it is speculated that *Bupleurum falcatum* L., which has the efficacy of “removing heat” and “resolving masses”, may alleviate cancer clusters. Therefore, SSD was selected from the compounds of this herb because there have been no prior studies on its effects on CRC. Numerous studies have reported the anticancer effects of SSD. It suppresses the survival of undifferentiated human thyroid carcinoma cells by increasing apoptosis and cell cycle arrest [35]. It also inhibits the development of hepatocellular carcinoma cells (HCCs) and downregulates syndecan-2, matrix metalloproteinase (MMP)-2, MMP-13, and tissue inhibitor of metalloproteinase-2 expression in rat HCC liver tissues [36].

Experiments were performed to confirm the suppressive effect of SSD on CRC cells. When SSD was administered to RAW 264.7 cells, cytotoxicity was not observed up to 50 μM [23]. Moreover, according to several studies, the anti-tumor effect of SSD is set at a maximum concentration of 10 μM [28,30,37,38]. Based on these studies, the concentrations used in this study were set to 2.5–10 μM. When four different metastatic CRC cell lines were treated with SSD, we observed a marked decline in cell survival rates (Figure 1A–D). Colony formation ability was also confirmed in CT26 and HCT116 cells (Figure 1E,F). These results showed that SSD suppressed the proliferation and colony formation abilities of CRC cells in a dose-dependent manner.

Autophagy is the cellular process of the self-elimination of unnecessary or dysfunctional components that are separated into autophagosomes [16]. The autophagosome then combines with a lysosome to form an autolysosome [18]. LC3B plays a role in autophagosome formation by expanding the autophagosome membrane. p62, also known as SQSTM1, binds to ubiquitinated proteins and attaches to LC3B [39]. The p62-bound ubiquitinated substrates are degraded in autophagosomes [39]. Increased p62 levels indicate impaired autophagy caused by dysfunctional autolysosome formation, which can result in cell death [40]. In this study, the induction of autophagy was observed by detecting the translocation of LC3 and increased mRNA and protein levels of LC3B and p62.

Apoptosis is one of the main factors related to the inhibition of cancer cells and involves both intrinsic and extrinsic pathways [7]. The intrinsic pathway is initiated in the mitochondria. Bcl-2 and Bcl-xL present on the mitochondrial membrane regulate the release of cytochrome C [8,9]. The released cytochrome C binds to Apaf-1 and procaspase-9 to form apoptosomes [41]. Through this process, caspase-3 is activated and apoptosis occurs [12]. PARP, which repairs damaged DNA, is cleaved by caspase-3 and induces apoptosis [15]. The extrinsic pathway originates from the binding of death ligands to receptors, which cleaves caspase-8 and subsequently caspase-3 and PARP [13,14]. In this study, SSD downregulated Bcl-2 and Bcl-xL, leading to the upregulation of cytochrome C release. Consequently, the cleavage of caspase-9, caspase-3, and PARP increased. Elevated mitochondrial depolarization and an increase in apoptotic cells were detected. Thus, SSD-induced apoptosis occurs through both intrinsic and extrinsic pathways.

The MAPK signaling pathway is closely associated with autophagy and apoptosis [42,43]. Piperlongumine mediates autophagy by activating p38 MAPK in U20 cells [44]. Moreover, p38 activation phosphorylates BimEL, leading to the apoptosis of PC12 cells [45]. The autophagy of MCF-7 breast cancer cells with TNF is induced via the ERK signaling pathway [46]. Additionally, ERK activation promotes apoptosis by caspase-8 activation, Bcl-2 family downregulation, and cytochrome C release [47,48]. In this study, p38 and ERK phosphorylation were observed after SSD treatment. Based on these results, SSD may induce autophagy and apoptosis by targeting the p38 and ERK signaling pathways.

The proliferation of HSVtk/HEP3B hepatocellular cancer cells was inhibited by the intraperitoneal administration of SSD (10 mg/kg) for one week in BALB/c nude mice that were subcutaneously injected with the cells [49]. Another study showed that the number and size of thyroid cancers were reduced by the intragastric administration of SSD (5–20 mg/kg) in athymic nude mice for 14 days [35]. The oral gavage of SSD (300 mg/kg) in ICR mice for one week resulted in hepatotoxicity through hepatocyte apoptotic death [50]. Based on these studies, the highest concentration for oral administration was set at 20 mg/kg for 14 days in the in vivo experiment. Serum analysis revealed that hepatic and renal toxicities caused by SSD were not observed in this study. The number of pulmonary tumor nodules was lowest in the 5-FU group but also exhibited a considerable decrease in the 10 and 20 mg/kg groups compared with the control group. A significant reduction in the number of nodules was observed at a low concentration (5 mg/kg). Based on the results of Western blot analysis for autophagy- and apoptosis-related factors in the lung tissue, elevations in LC3B and p62 were observed after SSD treatment. Moreover, SSD reduced Bcl-2 and Bcl-xL expression and increased cytochrome C expression. The cleavage of caspase-3, caspase-8, caspase-9, and PARP also increased compared with that in the control group. Overall, the oral administration of SSD inhibited the lung metastasis of CRC by reducing the number of tumor nodules through autophagy and apoptosis.

## 5. Conclusions

This study showed that SSD suppressed the survival and lung metastasis of CRC cells through in vitro and in vivo experiments. SSD interfered with the proliferation and metastasis of CRC cells in the lungs by inducing autophagy and apoptosis via p38 and ERK activation. In conclusion, these results suggest that SSD might be a novel therapeutic option for the treatment of metastatic CRC.

## Figures and Tables

**Table 1:** Serological analysis results of AST, ALT, creatinine, and BUN after the animal experiment.

	SSD (mg/kg)				5-FU (10 mg/kg)
	0	5	10	20	
AST (IU/L)	128.8 ± 85.41	148.86 ± 56.67	185.60 ± 39.30	134.86 ± 62.6	132.29 ± 40.72
ALT (IU/L)	23.83 ± 3.97	25.00 ± 4.16	28.00 ± 5.70	26.86 ± 6.64	23.71 ± 5.02
BUN (mg/dL)	19.33 ± 2.58	19.71 ± 4.75	21.50 ± 2.51	17.00 ± 1.51	20.86 ± 1.77
Creatinine (mg/dL)	0.20 ± 0.04	0.18 ± 0.03	0.19 ± 0.03	0.18 ± 0.02	0.18 ± 0.03

## Data Availability

The original contributions presented in the study are included in this article, and further inquiries can be directed to the corresponding authors.

## Supplementary Materials

The following supporting information can be downloaded at: https://www.mdpi.com/article/10.3390/nu16121844/s1, Figure S1: Effect of SSD on the proliferation of mouse primary bone marrow macrophages. Cell viability of SSD-treated mouse bone marrow macrophages for 24 h was determined using the WST reagent; Table S1: DNA oligo sequences.
