# Supplementary material for: Saikosaponin D Inhibits Lung Metastasis of Colorectal Cancer Cells by Inducing Autophagy and Apoptosis
Source: Nutrients. 2024 Jun 12;16(12):1844. doi: 10.3390/nu16121844 (PMC11206761; doi:10.3390/nu16121844)
Supplement: Supplementary file 1 [file nutrients-16-01844-s001.zip › nutrients-3039649-supplementary.pdf]

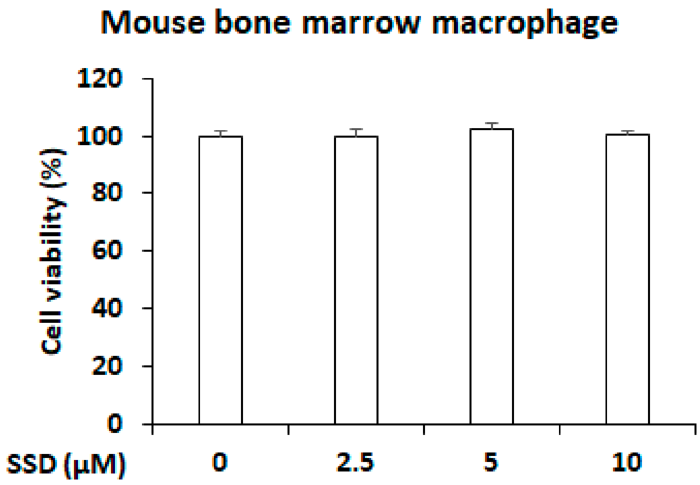

Figure S1. Effect of SSD on the proliferation of mouse primary bone marrow macrophages. Cell viability of SSD-treated mouse bone marrow macrophages for 24 h was determined using the WST reagent.

Culture of Bone Marrow-Derived Macrophages

Bone marrow cells (BMCs) were isolated from femurs and tibiae's bone marrow in male ICR mice (5-week old). Cells were cultured in α-MEM plus 10% FBS, 1% penicillin-streptomycin, and 10 ng/mL macrophage colony-stimulating factor (M-CSF) for 24 h. Suspension cells were harvested and seeded in 10 cm culture dish with M-CSF (30 ng/mL) for 3 days. Adherent cells were collected as bone marrow-derived macrophages. It has been described in Supplemental methods section.

Table S1. DNA oligo sequences

Name	Forward primer (5' to 3')	Reverse primer (5' to 3')
Mouse LC3B	GACGGCTTCCTGTACATGGTTT	TGGAGTCTTACACAGCCATTGC
Mouse p62	GCTGCCCTATACCCACATCT	CGCCTTCATCCGAGAAAC
Mouse GAPDH	AGCCTCGTCCCGTAGACAAA	CCTTGACTGTGCCGTTGAAT
Human LC3B	GAGAAGCAGCTTCCTGTTCTGG	GTGTCCGTTCACCAACAGGAAG
Human p62	GCACCCCAATGTGATCTGC	CGCTACACAAGTCGTAGTCTGG
Human GAPDH	CATGAGAAGTATGACAACAGCCT	AGTCCTTCCACGATACCAAAGT
